# A Multidisciplinary Approach to Diagnosing Blastic Plasmacytoid Dendritic Cell Neoplasm (BPDCN): Practical Recommendations and Insights from Countries of the Gulf Cooperation Council

**DOI:** 10.3390/cancers17020221

**Published:** 2025-01-11

**Authors:** Nasir Bakshi, Ayman Al Hejazi, Hatim Al-Maghraby, Areej Al Mugairi, Ahmad S. Alotaibi, Haitham Khogeer, Rania Medhat Seliem, Ramesh Pandita, Heba Raslan, Phyu P. Aung, Robert S. Ohgami

**Affiliations:** 1 Department of Pathology and Laboratory Medicine, King Faisal Specialist Hospital and Research Centre, Riyadh 12713, Saudi Arabia; nbakshi@kfshrc.edu.sa (N.B.); hkhogeer97@kfshrc.edu.sa (H.K.); 2 Department of Oncology, King Abdulaziz Medical City, Ministry of National Guard—Health Affairs, Riyadh 11426, Saudi Arabia; hejazia@mngha.med.sa; 3 Department of Pathology and Laboratory Medicine, King Faisal Specialist Hospital and Research Center, Madinah 42522, Saudi Arabia; drpathology@gmail.com; 4 Hematopathology Division, Department of Pathology and Laboratory Medicine, King Abdulaziz Medical City, Riyadh 14611, Saudi Arabia; moghairia@mngha.med.sa; 5 Oncology Centre, King Faisal Specialist Hospital and Research Centre, Riyadh 12713, Saudi Arabia; aalotaibi97@kfshrc.edu.sa; 6 Rashid Hospital, Dubai Health Authority, Dubai P.O. Box 4545, United Arab Emirates; rmseliem@dubaihealth.ae; 7 Department of Hematology, Kuwait Cancer Control Centre, Shuwaikh 42262, Kuwait; ramesh.pandita@gmail.com; 8 Department of Pathology and Laboratory Medicine, King Fahad Specialist Hospital, Dammam 32253, Saudi Arabia; hebaraslan2012@gmail.com; 9 Department of Anatomic Pathology, MD Anderson Cancer Center, The University of Texas, Houston, TX 77030, USA; paung@mdanderson.org; 10 ARUP Laboratories, Department of Pathology, University of Utah, Salt Lake City, UT 84103, USA

**Keywords:** blastic plasmacytoid dendritic cell neoplasm, tagraxofusp, Gulf Cooperation Council, dendritic cells/pathology, hematologic neoplasms/diagnosis, consensus recommendations, differential diagnosis

## Abstract

Blastic plasmacytoid dendritic cell neoplasm (BPDCN) is an aggressive orphan hematopoietic malignancy characterized by cutaneous and systemic hematologic involvement. BPDCN is frequently misidentified, but early, accurate diagnosis is critical to extending patient survival using tagraxofusp, a first-in-class CD123-targeted therapy, and increasing their chances of receiving a potentially curative stem cell transplantation. Cases of BPDCN in countries of the Gulf Cooperation Council are lower than the extrapolated incidence from other geographic locations due to lack of awareness of key diagnostic features, lack of consensus on the minimal phenotype for diagnosis, and lack of local immunohistochemistry testing facilities, contributing to underdiagnosis in this region. Practical recommendations, a streamlined diagnostic panel, and suggested multidisciplinary approaches based on expert experience regarding diagnostic and clinical challenges specific to this region, and a review of the literature are presented here to facilitate diagnosis of BPDCN in this region by primary care physicians, dermatologists, and hematologists.

## Simple Summary

Blastic plasmacytoid dendritic cell neoplasm (BPDCN) is an aggressive cancer affecting the skin, blood, bone marrow, and lymph nodes. It occurs more often in older men. It is difficult to diagnose this condition because it is rare and has many possible symptoms. Early, accurate diagnosis is important, so patients can receive the only available treatment, tagraxofusp, to help extend their survival until stem cell transplantation, which may cure the disease. Fewer cases of BPDCN have been diagnosed in the countries of the Gulf Cooperation Council (GCC) than expected because doctors may be unfamiliar with BPDCN, and there is currently no agreement on what set of blood markers can be used to diagnose this disease. A panel of experts who know this region reviewed the literature and developed practical recommendations to help doctors diagnose BPDCN quickly and accurately in the GCC.

## 1. Introduction

Blastic plasmacytoid dendritic cell neoplasm (BPDCN) is an orphan hematologic malignancy arising from plasmacytoid dendritic cells (pDC) [1]. Globally, BPDCN is reported to account for 0.44% of all hematologic malignancies each year, which is around 700 cases in the USA and 1000 in Europe [2,3]. BPDCN occurs in all races and all geographic locations. It has been described in all age groups (including children) but is most common in people aged 60 years and over [2,4,5]. The condition is 2–4-fold more common in males than females, with male predominance increasing with age at diagnosis [2,4,5].

BPDCN was previously known as CD4+ CD56+ cutaneous lymphoma, blastic natural killer cell lymphoma/leukemia, agranular CD4+ natural killer cell leukemia, and agranular CD4+ CD56+ hematodermic neoplasm [6,7,8]. The discovery that BPDCN originates from plasmacytoid dendritic cells and type 2 dendritic cells—which also give rise to acute myeloid leukemia (AML), myelodysplastic syndromes (MDS), and myeloproliferative neoplasms (MPN)—led to the change in its nomenclature.

The condition was renamed ‘BPDCN’ in the 4th edition of the *WHO Classification of Myeloid Neoplasms and Acute Leukemia*. At that time, BPDCN was included as a member of the ‘AML and related neoplasms’ subclass, but was reclassified as a subgroup of myeloid malignancies in its own right in the 2016 revision to the 4th edition [9]. The *International Consensus Classification (ICC) of Myeloid Neoplasms and Acute Leukemias*, published in 2022, also uses this classification [10].

In the 5th edition of the WHO guidelines, BPDCN was further reclassified under histiocytic/dendritic cell neoplasms and pDC neoplasms [11]. This class also includes mature plasmacytoid dendritic cell proliferation (MPDCP), which is associated with myeloid neoplasms—most often chronic myelomonocytic leukemia (CMML), but also MDS, MPN, and pDC-AML (a form of AML with monocytic differentiation)—that do not express CD56 [1,12,13,14]. In contrast, the ICC categorizes BPDCN under myeloid neoplasms, highlighting its clinical and molecular similarities with other myeloid malignancies. Both frameworks recognize BPDCN as a distinct entity but emphasize different aspects of its biology: the WHO focuses on its histiocytic and dendritic cell lineage, whereas the ICC underscores its shared features with myeloid neoplasms. Together, these perspectives provide a comprehensive and complementary understanding of BPDCN’s complex biology, enhancing its diagnosis and informing treatment strategies.

A detailed overview of the pathogenesis and molecular etiology of BPDCN is outside the scope of this paper; however, several groups have covered these topics in detail [2,13,15,16,17,18,19,20,21,22].

BPDCN is a highly aggressive malignancy that had a poor prognosis prior to the approval of tagraxofusp [23]. The introduction of tagraxofusp, a first-in-class CD123-targeted therapy, has provided a treatment that can extend patients’ survival beyond 12 months [24,25]. This survival improvement will allow more patients to bridge to alloSCT, the only potential curative treatment presently available. However, improved patient outcomes depend on correctly diagnosing BPDCN, so patients are treated with correct, effective treatment.

However, BPDCN is frequently misdiagnosed as other hematological malignancies, including AML, histiocytic/dendritic cell neoplasms, and natural killer/T-cell lymphomas, due to heterogeneity of presentation, changing nomenclature, and a lack of precise guidelines [2,4]. BPDCN may present alone or in association with other hematologic malignancies including MDS, CML, CMML, and AML. The presentation is heterogenous. Patients may have skin lesions with or without pancytopenia at diagnosis, and the immunophenotype is variable. The skin lesions themselves vary in their appearance and may include asymptomatic single or multiple skin nodules, plaques or purpuric lesions possibly associated with erythema, and hyperpigmentation, purpura or ulceration [16,17,19,20].

The publication of the 5th edition of the *WHO Classification Of Myeloid Neoplasms And Acute Leukemia* and current targeted therapies provide opportunities to re-examine and re-evaluate diagnostic standards for BPDCN. This is likely to change with the recently introduced guidelines and the increasing availability and use of immunophenotyping in diagnosis [1]. However, these guidelines are global in scope and do not take into account potential regional variations in experience and resources available. To aid health care providers in the countries of the Gulf Cooperation Council (GCC), this manuscript was developed by an international expert panel who reviewed the published clinical literature regarding BPDCN and applied their experience with this condition in the countries in the GCC to provide revised practical recommendations for its clinical diagnosis that are specific to this region.

### BPDCN in the Countries of the GCC

**Figure 1:**
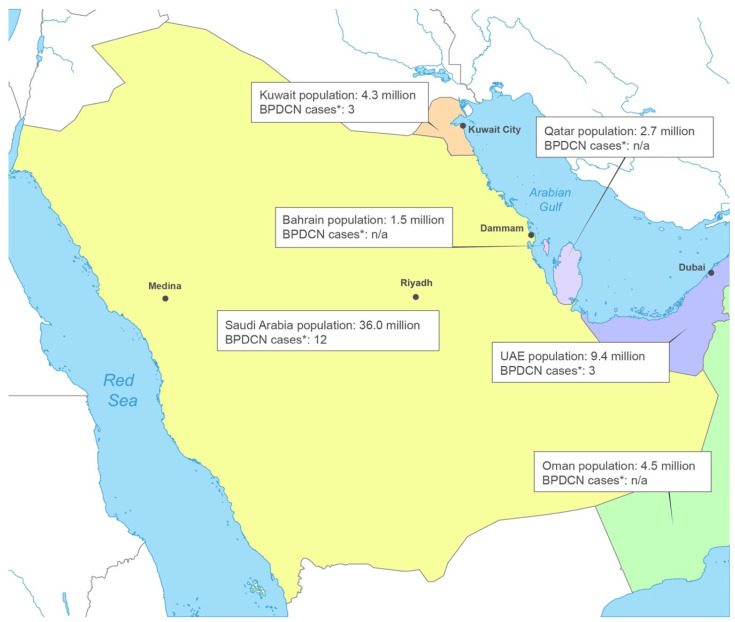
Map of the Countries in the Gulf Cooperation Council. National population figures as of 2021 [23] and the number of cases diagnosed in past 5 years are shown. • Location of represented hospitals. * n/a = not applicable.

The GCC (Figure 1) includes Saudi Arabia, the United Arab Emirates (UAE), Oman, Kuwait, Qatar, and Bahrain. Hospitals with which the authors are affiliated are shown, including four hospitals in Saudi Arabia (two in Riyadh, and one each in Medina and Dammam), one hospital in Dubai, the United Arab Emirates, and one hospital in Kuwait City, Kuwait.

In the last 5 years, 12 patients were diagnosed with BPDCN at the four hospitals in Saudi Arabia, while three patients were diagnosed at the hospital in Kuwait and three more at the hospital in the UAE. Among the patients diagnosed with BPDCN in Saudi Arabia, almost all were male; this is a greater predominance than what is seen globally. The majority of these patients were aged 65–67 years at diagnosis, which is consistent with the known bimodal age distribution of BPDCN, with peaks in people under 20 or over 60 years old [26].

It is common in the GCC for dermatologists to refer suspected cases of BPDCN, possibly because the skin lesions are relatively easy to identify by patients and health care providers, which may lead to early diagnosis. A thorough evaluation of any skin lesions, both by history and physical examination, is highly recommended. Although a small discoloration or patchy lesion may be ignored, especially in unexposed areas, an advanced skin lesion could in fact be infiltrating soft tissue or even bone. The majority of the remainder of patients are referred by hematologists. Only rarely is a patient referred by a general practitioner (GP). Approximately 50% of patients present with skin lesions only; the remainder also have signs and symptoms of leukemia.

The small number of patients in the GCC makes it impossible to generalize about signs and symptoms of BPDCN unique to patients in the GCC. It also is not possible to draw conclusions about differences in presentation and prognosis in different subpopulations (e.g., different age groups and the nature of the initial presentation).

There is no official estimate of the incidence of BPDCN in the Middle East; but, based on the estimated global incidence, approximately 25 cases per year would be expected throughout the region as compared to the number diagnosed according to review of records (16 cases/5 years = 3.2 cases/year).

The reasons identified for underdiagnosis of BPDCN in the GCC include: lack of awareness of its key diagnostic features (many patients are referred with a suspicion of diseases other than BPDCN); BPDCN misdiagnosed as AML (especially monocytic AML); heterogenous disease presentation; lack of consensus on the minimal phenotype to establish the diagnosis of BPDCN; and the need for some centers to send immunohistochemistry samples to external international laboratories (with the associated delays).

Few practitioners involved in the diagnosis of BPDCN in the GCC have access to specialist training. Where training is available, it is usually only aimed at hematopathologists.

## 2. Presentation and Prognosis of BPDCN

BPDCN may present alone or in association with other hematologic malignancies including MDS, CMML, and AML. Most patients present with a combination of dermatologic signs and symptoms and pancytopenia. A minority of patients present with pancytopenia only; these patients are at high risk of misdiagnosis if the findings of histological staining and immunophenotyping are not evaluated and interpreted accurately [27].

The appearance of skin lesions is heterogenous; they may be asymptomatic single or multiple skin nodules, plaques or bruise-like lesions possibly associated with erythema, and hyperpigmentation, purpura or ulceration [16,17,19,20].

BPDCN rarely manifests with involvement of the bone marrow exclusively (with an absence of cutaneous manifestations). However, skin lesions may emerge later during the disease course or during disease relapse. Splenomegaly and lymphadenopathies are common.

The disease course is aggressive; progression is marked by increasing skin involvement, progressive cytopenia, and involvement of the bone marrow, lymph nodes, and/or viscera and the central nervous system (CNS) [16,17,19,20]. The historical prognosis of BPDCN, prior to the approval of tagraxofusp, is poor, with a median overall survival (OS) of 8–14 months [3,28,29,30]. There is no BPDCN clinical staging system.

## 3. Diagnosis of BPDCN

Until recently, there were no disease-specific guidelines for the diagnosis and treatment of BPDCN (although, several have now been published [31,32]), and the literature on BPDCN was mostly based on case reports with no substantive reviews of treatment and outcomes.

Biopsies are critical for the evaluation of cellularity and lineage, to exclude lesions associated with non-leukemic pathologies, and to provide samples for immunophenotyping. Hematoxylin and eosin (H&E) stains are generally recommended for histological analysis, although May–Grünwald–Giemsa stains are preferred for bone marrow aspirates.

According to the 5th edition of the WHO guidelines, BPDCN is defined by immuno-positivity for three core biomarkers, CD123, CD4 and CD56, in addition to one of the following: TCF4, TCL1, CD303, and CD304. A small subset of cases may express only two of the three primary markers, CD123, CD4, and CD56. The guidelines offer an alternative definition: positivity for three or more of CD123, TCF4, TCL1, CD303, and CD304 with the absence of CD3, CD14, CD19, CD34, lysozyme, and myeloperoxidase. In clinical practice, the triad of CD4, CD56, and CD123 positivity and the absence of B-, T-, and myeloid lineage-specific markers should prompt clinicians to consider BPDCN and encourage further work-up [27].

Although an abnormal karyotype has been reported in most BPDCN cases, cytogenetics and molecular testing currently do not play a major role in the diagnosis of BPDCN. In part, this is because there is no specific cytogenetic abnormality that is diagnostic for the disease. Around 85% of patients have at least one mutation, while 30–45% have at least three [27]. In the future, there may be a role for cytogenetics in both differential diagnosis and therapeutic response assessments.

## 4. Proposed Diagnostic Pathways

**Figure 2:**
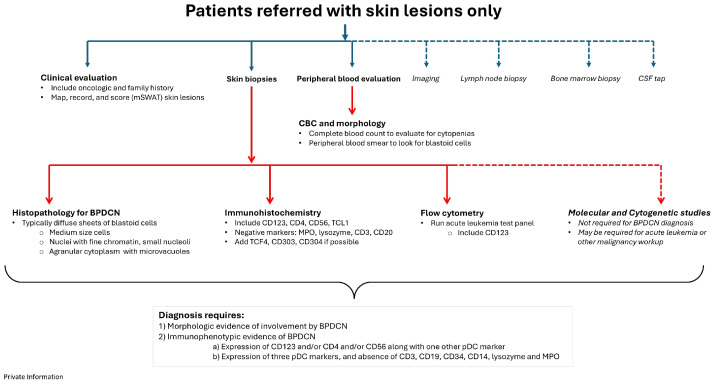
Proposed BPDCN diagnostic pathway for patients referred with skin lesions only. Required and strongly recommended options are shown with the solid arrows, while dashed arrows show ancillary studies that are not always required but may be useful.

**Figure 3:**
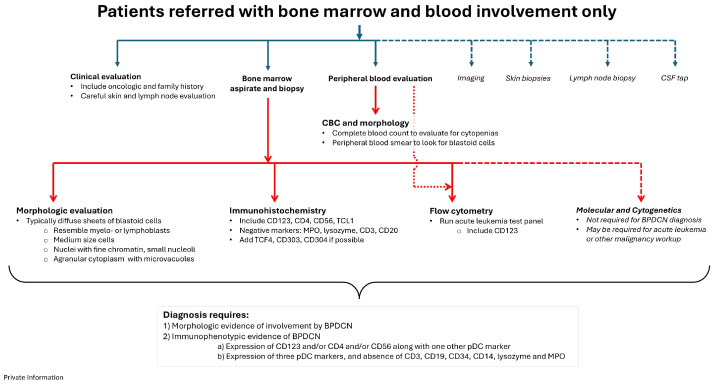
Proposed BPDCN diagnostic pathway for patients referred with bone marrow and blood involvement only. Required and strongly recommended options are shown with the solid arrows, while dashed arrows show ancillary studies that are not always required but may be useful.

**Figure 4:**
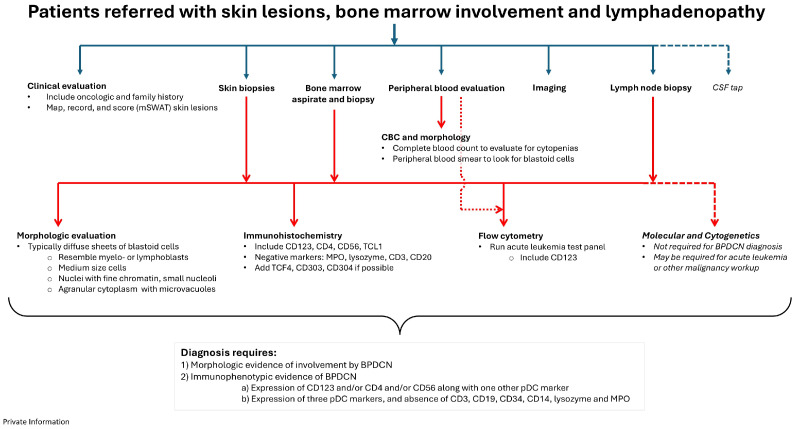
Proposed BPDCN diagnostic pathway for patients referred with skin lesions, bone marrow involvement, and lymphadenopathy. Required and strongly recommended options are shown with the solid arrows, while dashed arrows show ancillary studies that are not always required but may be useful.

Based on the WHO recommendations (5th edition) and clinical experience in the GCC, the following three interlinking diagnostic flow charts, impactful for the GCC and beyond, are proposed:

1. Patients referred with skin lesions only (Figure 2);
2. Patients referred with blood/bone marrow involvement only (Figure 3);
3. Patients referred with skin lesions, bone marrow involvement, and lymphadenopathy (Figure 4).

For all diagnostic pathways, a multidisciplinary team including general clinical practitioners and subspeciality experts in clinical and pathology disciplines is recommended for diagnosis, treatment, and continued care.

### 4.1. Patients with Skin Lesions

Figure 2 shows the proposed diagnostic pathway for patients who are referred with skin involvement only. During the clinical evaluation, involvement can be quantified using the modified Severity-Weighted Assessment Tool (mSWAT). mSWAT is calculated by multiplying the area covered with lesions by the number of lesions to assign a severity score that is useful for monitoring the patient’s progress [27].

It is recommended that multiple (at least two) 4–5 mm-deep skin punch biopsies from the skin and subcutaneous tissue are taken. These should be representative of the lesion and contain sufficient viable tumor cells for multiple histological and immunophenotyping tests and for other ancillary studies such as karyotyping and next generation sequencing. Crushing and/or hemorrhagic artifacts should be avoided. Fine needle aspiration biopsy is an alternative method of obtaining material for flow cytometry if obtaining a tissue biopsy proves difficult.

If there is occult bone marrow involvement, the complete blood count (CBC) results may provide the first indication; patients with hemoglobin <10 g/dL, leukocyte count <4 × 10^9^/L, and platelets <150 × 10^9^/L should undergo a bone marrow biopsy, and the results ideally should be reviewed by a hematopathologist [33].

Complaints of headache, dizziness, seizures, vision problems, facial nerve paralysis, paresthesias, or back pain that might indicate CNS involvement should prompt a cerebrospinal fluid (CSF) tap, ideally with flow cytometry testing. If lymphadenopathy is later identified, surgical excisional biopsies are best performed and assessed by flow cytometry along with routine morphologic assessment and immunohistochemistry.

### 4.2. Patients with Bone Marrow and Blood Involvement

For patients who are referred due to presumed bone marrow and blood involvement only, history taking should mirror that conducted for patients with skin lesions, and any suspicious skin lesions should be biopsied (Figure 3). Evaluation of the peripheral blood and a bone marrow biopsy is required to establish the extent of disease involvement and provide a baseline to assess therapeutic response. The bone marrow aspirate and biopsy typically show sheets of blastoid cells, though scant interstitial involvement can occur; neoplastic cells will be more clearly highlighted by immunohistochemical stains. Flow cytometry should be performed on the bone marrow sample when possible.

BPDCN patients are at high risk of being misdiagnosed, especially if there is a lack of clarity in the immunophenotype. Further development of skin lesions at any point should prompt additional investigations, especially skin biopsies. The primary differential diagnosis for patients without skin lesions includes other predominantly circulating hematologic malignancies such as AML, T-ALL and circulating NK-cell or T-cell lymphomas.

### 4.3. Patients with Skin, Bone Marrow, and Lymph Node Involvement

For patients who are referred due to involvement of skin, marrow and lymph nodes, the guidance given above for history taking, assessment, and differential diagnosis of patients also apply here (Figure 4). The lymph node biopsy should have immunophenotyping performed that includes immunohistochemistry and/or flow cytometry. In the setting of patients with lymphadenopathy, imaging studies to assess for extent of lymph node and other tissue involvement is important. Radiologic imaging also provides a baseline to monitor disease involvement following treatment.

These patients with generalized advanced systemic involvement have the advantage of beginning the diagnostic process with an unusual combination of signs and symptoms that might prompt the early involvement of a multidisciplinary team and early willingness to consider a less common diagnosis.

## 5. Practical Recommendations for Diagnosis of BPDCN in the GCC

Patients presenting with skin lesions with unusual or atypical features of common dermatoses (especially if combined with symptoms of pancytopenia) should be referred for dermatopathological and hematological investigations. Patients with signs and symptoms of pancytopenia without skin lesions would usually be referred to a hematologist for evaluation. It can be beneficial to review the results of biopsies and other laboratory results with a multidisciplinary team that includes general pathologists, hematopathologists, and dermatopathologists to make an accurate and timely diagnosis. This is due to the breadth and complexity of the differential diagnosis. Greater awareness of BPDCN among GPs, community dermatologists, and other non-specialists could increase the numbers of referrals of patients to a specialist dermatologist.

### 5.1. Sample Collection

All patients presenting with skin lesions should have skin biopsies, and all patients with signs and symptoms of pancytopenia should have peripheral blood collected, as well as bone marrow aspirate and biopsy. Dermatopathologists and/or hematopathologists receiving samples from patients with suspected BPDCN should also receive a detailed history from the referring clinician that should include reports of prior hematological malignancies (ALL, MDS, MPN, myeloma), biopsies, and CBC reports. The referring clinician should also provide detailed descriptions of skin lesions, ideally with photographs.

Peripheral blood should be collected in EDTA tubes and bone marrow aspirate and biopsy in heparin tubes. In the event of a dry tap, clinicians may submit an additional bone marrow biopsy specimen in normal saline for processing within a maximum timeframe of 24 h. Skin and other soft tissue biopsies should be submitted in 10% buffered formalin solution for routine histologic examination and immunohistochemical studies.

Samples being submitted for flow cytometric studies ideally should be submitted in RPMI, but samples submitted in heparin or EDTA are also adequate. Immunophenotyping and immunohistochemistry should be performed in a laboratory which is certified by an accreditation program such as the College of American Pathologists (CAP) accreditation program on clinically validated instruments with clinically validated reagents.

### 5.2. Patient Examinations

The physical exam should include careful assessment of lymph nodes: if lymphadenopathies are detected, biopsies should be ordered. A lumbar puncture should be ordered if CNS involvement is suspected. Patients should also be assessed for signs and symptoms of splenomegaly and indications of upper airway or digestive tract involvement to provide additional information for the differential diagnosis.

The histologic staining of BPDCN biopsy samples usually reveals medium sized blastoid appearing cells resembling lymphoblasts or myeloblasts. The nuclei have irregular contours, fine chromatin, and one or more small nucleoli. The cytoplasm is usually scanty, blueish (i.e., basophilic), and agranular, and may show vacuoles that sometimes merge under the cytoplasmic membrane, showing a string-of-pearls pattern. BPDCN cells show tail-like projections. Bone marrow biopsies may show infiltration in diffuse and/or interstitial patterns, usually with encroachment upon all other hematopoietic marrow elements. In skin biopsies, sheets of immature or primitive-looking large cells in the upper and deep dermis are seen.

In the GCC, not all centers have the laboratory facilities to conduct the full range of immunohistochemical analyses recommended by the WHO. Consequently, we recommend a minimum positive marker panel of CD123, CD4, CD56, and TCL1 with the recommendation to additionally perform staining for other pDC lineage antigens including CD303, CD304, and TCF4, if possible (Table 1). In fact, co-expression of TCF4 and CD123 in tumor cells has a reported analytic sensitivity of 100% and a specificity of 99.8% for diagnosis of BPDCN [26]. Some centers will need to rely on external laboratories to conduct these tests. Finally, markers that are consistently negative in BPDCN are lysozyme, MPO, CD20, CD19, CD14, CD34, and CD3; any expression of these markers is indicative of another malignancy.

Where available, flow cytometry is important, as the information it provides is often more sensitive and specific, especially regarding the bright expression of BPDCN markers. To align with the testing protocols in the GCC, 2-step flow cytometry consisting of a wide initial acute leukemia panel, followed by further investigations for specific markers if the initial panel is inconclusive, is recommended. Testing consortia, such as EuroFlow (euroflow.org; accessed 1 February 2024), can help supply markers and knowledge to help with flow cytometry testing. It is often helpful to incorporate immunohistochemical staining when assessing possible cases of BPDCN because TCL-1, TCF-4, and CD303 are not assessable by flow cytometry. If flow cytometry is not available, immunohistochemical staining must be used; CD123, CD303, CD304, TCL1, TCF4, and TdT can all be adequately assessed with this method.

### 5.3. Differential Diagnosis

Heterogeneity in the presentation, morphology, and immunophenotype of BPDCN combine to make the differential diagnosis of this disease challenging. The breadth of the differential diagnosis means that patients may be evaluated by multiple specialists, require numerous laboratory tests, undergo several biopsies, and possibly diagnostic imaging before a final diagnosis is confirmed (Table 2).

BPDCN presents immunophenotypes that overlap substantially with other hematological malignancies (Table 2). Some immunophenotypic markers that are typically absent in BPDCN have been observed in a subset of cases due to the biological heterogeneity of the disease or the presence of co-existing immature plasmacytoid dendritic cells including CD34 and CD117 [25]. Although not considered typical markers of BPDCN, expression of CD2, CD7, and even CD79a have also rarely been reported [20,29]. Expression of the marker S-100 has been observed with other markers of plasmacytoid dendritic cells, primarily in pediatric patients [30]. BPDCN cells have also been reported to have variable expression of several markers including CD2 (19% positive), CD7 (64% positive), and CD38 (18% positive) [20].

This wide variability in immunophenotypic markers, even those considered characteristic of BPDCN, demonstrates the need for variant gating strategies for diagnosis. The challenge is to be able to differentiate MRD BPDCN from residual normal pDCs, including rare CD56+ reactive BPDCs.

## 6. Treatment and Monitoring

Historically, BPDCN was treated with intensive chemotherapy and, ideally, allogeneic stem cell transplantation (SCT), or alloSCT. As with other hematologic malignancies, a stem cell transplant is the only potential cure for BPDCN. No chemotherapy is approved for use in BPDCN, and although initial responses are frequently good (with remission rates ranging from 36% to 60%), patients often relapse, and OS is generally less than 12 months [16,17,19,20,41,42]. Chemotherapy is therefore of limited usefulness as a bridge to alloSCT, highlighting the need for new treatments options with better efficacy in BPDCN, especially more durable responses that will facilitate a higher rate of success in bridging patients to transplant, as well as a better safety profile.

As discussed above, all BPDCN cells overexpress CD123, and CD123 is therefore an appealing target for treating this malignancy. The CD123-directed therapy tagraxofusp (tagraxofusp-erzs; Elzonris™) is the first, and currently the only, approved targeted treatment for BPDCN and consists of a recombinant fusion protein consisting of human interleukin-3 conjugated to a truncated diphtheria toxin payload. Tagraxofusp was initially approved in the USA in 2018 to treat all patients with BPDCN, including adults and pediatric patients ≥ 2 years old [43], and it has since also been approved in Europe for first-line treatment of BPDCN in adults ≥ 18 years old [44]. In patients receiving first-line treatment in the phase 2 pivotal 0114 study (NCT02113982), the median OS was 15.8 months (95% CI: 9.7–25.8) with a median follow up duration of 34 months [42]. The rate of complete response (CR) plus clinical CR (CRc; CR with residual skin abnormality not indicative of active disease) was 57%, and the median duration of CR + CRc was 24.9 months (95% CI: 3.8–not reached). In addition, 51% of first-line patients who achieved CR + CRc bridged to SCT, and the median OS in those patients was 38.4 months (range 3.4–58.1), with 72% remaining in remission for ≥12 months after transplant [42]. Treatment-emergent adverse events (AEs) of grade ≥ 3 occurred in 75 of 89 patients (84%) enrolled in the trial, including 84 who received targaxofusp at a dose of 12 µg/kg once daily (65 first-line; 19 relapsed/refractory). The most common grade ≥ 3 treatment-emergent AEs were thrombocytopenia (29; 33%), increased alanine aminotransferase (28; 32%), and increased aspartate aminotransferase (27; 30%). Most treatment-emergent AEs occurred in cycle 1, and myelosuppression was modest and reversible. Capillary leak syndrome (CLS) of any grade was observed in 18 patients (21%) who were treated with targaxofusp (12 µg/kg daily), which occurred primarily in cycle 1 and did not recur [42].

The available real-world evidence is similar, including a study of 22 European patients receiving first-line tagraxofusp, which reported a median OS of 20 months (95% CI: 10–not reached). The rate of CR was 67%, and the median duration of response (DOR) was 8.9 months (95% CI: 2.2–not reached) after over 2 years of follow up with no new safety signals [24]. This included a median OS of 11 months in 11 patients who were not transplanted, while in the 11 patients (50%) who bridged to SCT, the median OS was not yet reached. Likewise, 18 real-world European patients with relapsed or refractory BPDCN had a median OS of 8.6 months (95% CI: 3.6–not estimable) and no new safety signals [45]. In 15 patients with ≥1 tumor assessment, 40% achieved CR, and the median DOR of all responses was 5 months (95% CI: 3–not estimable). Of these, six patients bridged to SCT and had durable post-transplant responses, including 83% who achieved CR and 17% who achieved partial response (PR), with the median OS not yet reached. The rates of patients who were bridged to SCT in these real-world studies, including 50% among treatment-naïve patients and 40% in those with relapsed or refractory BPDCN [24,45], demonstrate the benefit of tagraxofusp as a bridging therapy. In both real-world analyses, the majority of CLS events were mild, with no observed grade 4 or 5 CLS events, and most occurred in cycle 1.

In the future, CD123-targeted agents combined with chemotherapy (such as hyperfractionated cyclophosphamide, vincristine, doxorubicin, or dexamethasone [HCVAD]) and prophylactic CNS-directed treatment (such as alternating intrathecal methotrexate and cytarabine) may further improve outcomes for patients with BPDCN [46]. In addition, BCL-2 has been shown to be overexpressed in BPDCN [47], and deregulation of epigenetic regulation is a hallmark of these cells [48], suggesting possible roles for BCL-2 inhibitors and hypomethylating agents, including a sequential regimen of tagraxofusp followed by a combination of venetoclax and azacitidine [49].

The nature of BPDCN requires monitoring for disease progression to anticipate the possibility for patients with only skin symptoms at presentation of developing cytopenia (and vice versa). Once the patient has been diagnosed and has started treatment, their progress should be assessed through regular CBC, imaging, skin examinations, and monitoring for signs and symptoms of CNS involvement. In addition, expression of CD123 on BPDCN cells may decrease following treatment with anti-CD123 therapy such as tagraxofusp [50,51], which is similar to the reduction in CD20 expression observed in patients who receive the anti-CD20 antibody, rituximab [52].

## 7. Conclusions and Future Directions

There is a clear need for a practical diagnostic pathway that can be used by clinicians in the GCC and beyond. The reliance on biomarkers for the diagnosis of BPDCN (and other hematologic neoplasms) may be challenging in some settings. Recommendations for a minimum screening panel, including guidance for using immunohistochemistry as an alternative to flow cytometry if it is not readily available, as well as ongoing monitoring are summarized in Table 3.

The proposed diagnostic algorithms are based on the presence or absence of skin lesions or pancytopenia at presentation. In all cases, early biopsy is recommended to aid rapid diagnosis, and it is important for clinicians and pathologists to ensure proper ordering and submission of tissue samples, as well as to order the correct immunophenotyping/immunohistochemistry panel to help diagnose BPDCN cases as early as possible. The involvement of a multidisciplinary team with expertise in the diagnosis and management of dermatologic and hematologic diseases to review the results of the biopsies and to determine next steps is also highly recommended.

Finally, it is recommended that awareness of BPDCN be promoted among community dermatologists and GPs, including when to refer patients to a specialist, to help ensure that patients with BPDCN receive a prompt and accurate diagnosis.

Delivering a timely diagnosis of orphan diseases such as BPDCN is a major challenge for healthcare systems and individual clinicians. Despite access to state-of-the-art facilities and clinicians with extensive experience in the management of hematologic cancers, it seems likely that BPDCN is currently underdiagnosed in the GCC. These suggestions aim to help clinicians and pathologists to identify patients with BPDCN accurately and promptly and initiate appropriate treatment that will provide the best chances of achieving optimal patient outcomes.

## Figures and Tables

**Table 1:** At-a-glance immunophenotypic features of BPDCN and other overlapping entities.

Marker ^1^	BPDCN	AML	T−ALL	T−Cell Lymphoma
BCL−2	+	+/−	+/−	+
CD3	−	−	+	++
CD4	+	+/−	+/−	+/−
CD8	−	+/−	+/−	+/−
CD14	−	+/−	−	−
CD19	−	+/−	−	−
CD20	−	−	−	−
CD34	−	+/−	+/−	−
CD56	+	+/−	+/−	+/−
CD123	++	+/−	+/−	−
CD303	+	−	+/−	−
CD304	+	−	+/−	−
TCF4	++	−	−	−
TCL1	+	+/−	+/−	−
TdT	+/−	+/−	+/−	−
Lysozyme	−	+/−	−	−
Myeloperoxidase	−	+/−	−	−

^1^ CD3, CD4, CD14, CD19, CD34, CD56, CD123, CD303, CD304, TCF4, TCL1, lysozyme, and myeloperoxidase are recommended by WHO for diagnosis of BPDCN [1]. ++ (typically positive): marker serves as a hallmark feature of this disease entity. + (usually positive): marker is commonly positive in this disease entity. +/− (variable expression): marker expression is heterogeneous within this disease entity. − (usually negative or not expressed): marker is typically not present or is expressed at very low levels.

**Table 2:** Conditions that may be considered in the differential diagnosis of BPDCN.

Differential Diagnosis	Notes
Acute myeloid leukemia (AML) with monocytic differentiation (AML-M5)	Majority of blast cells from monocytic lineage. One-third of cases lack CD34. Cases are usually positive for lysozyme, often lack myeloperoxidase, and (weakly) co-express CD4 and/or CD123 and/or CD56 [34]
AML with plasmacytoid dendritic cell differentiation (pDC-AML)	Rare subtype of AML with ≥2% pDC expansion and interstitial distribution of pDCs intermixed with leukemic blasts. pDC may also be present in loose clusters. Positive for CD13, CD34, CD36, CD38, CD123, CD303, HLA-DR, and TCL1. Negative for CD56 and CD13. Frequently associated with a RUNX1 mutation [35]
Mature plasmacytoid dendritic cell proliferation associated with myeloid neoplasm (MPDCP)	Mature, fully differentiated blast cells. Positive for CD123, CD4, and HLA-DR. Negative for CD56, CD3, CD19, CD14, and MPO [13]
Chronic myelomonocytic leukemia (CMML)	Characterized by dysplastic bone marrow cells and persistent peripheral blood monocytosis. Two variants: dysplastic and proliferative. Splenomegaly is common and risk of transformation to AML is high. Cells are positive for CD34, CD33, CD117, CD123, and CD133. Negative for CD25, CD26, and CD38 [25]
Extranodal NK/T-cell lymphoma	Rare, disfiguring disease that can affect the upper respiratory and digestive tract, skin, or (rarely) bone marrow. Strongly associated with EBV infection of lymphoma cells. Cytolytic cells with cytotoxic granules including granzyme B, perforin, and TIA-1 or positivity for CD56 must be present. May also be positive for CD2, cytoplasmic CD3, and EBER [36]. Negative for CD123, CD4, and TCF4
T-cell prolymphocytic leukemia/lymphoma	Cells co-express CD3, CD4, and TCL1. A subset of cases may co-express CD56 [37]
Leukemia cutis	Usually associated with AML. Characterized by single or multiple firm papules, nodules, and plaques that may be skin-colored, red, brown, or purple. Expression of myeloperoxidase, CD15, CD43, CD45, CD34 (variable), CD68 (variable), and CD13 (variable) [38,39]
Subcutaneous panniculitis-like T-cell lymphoma	Rare cytotoxic T-cell lymphoma that exhibits leukemic infiltration of subcutaneous adipose tissue accompanied by a large number of macrophages. Cells are positive for CD2, CD3, CD7, CD8, and TIA-1. Negative for CD4, CD30, and CD56 [40]

EBV, Epstein–Barr virus; EBER, Epstein–Barr virus-encoded small RNAs.

**Table 3:** Summary of diagnostic recommendations.

Summary of Recommendations
Assemble multidisciplinary teams to review unusual cases that might be BPDCN and to manage patients with BPDCN once diagnosis is confirmed.
All patients should be biopsied with appropriate submission and handling of tissue for pathological examination, and the samples should undergo histochemical staining and immunophenotyping.
Where available, flow cytometry should be the first choice for immunophenotyping.
Two-step flow cytometry (an initial standardized panel followed by targeted biomarker screening) should be the norm.
As a minimum, CD123, CD4, CD56, and TCL1 should be assessed to diagnose BPDCN in addition to markers CD19, CD20, CD3, CD14, MPO, and lysozyme, which should be negative in BPDCN.
Careful monitoring of patients should detect additional signs and symptoms of BPDCN (e.g., development of skin lesions, pancytopenia, or CSF manifestations) that warrant further biopsies.
